# Prediction of stillbirth in women with overweight or obesity—A register-based cohort study

**DOI:** 10.1371/journal.pone.0206940

**Published:** 2018-11-19

**Authors:** H. Åmark, M. Westgren, M. Persson

**Affiliations:** 1 Department of Clinical Science and Education, Unit of Obstetrics and Gynecology, Karolinska Institute, Södersjukhuset, Stockholm, Sweden; 2 Department of Clinical Sciences, Intervention & Technology, Karolinska Institutet, Stockholm, Sweden; 3 Department of Medicine, Clinical Epidemiology Unit, Karolinska University Hospital, Stockholm, Sweden; Univesity of Iowa, UNITED STATES

## Abstract

**Objective:**

To develop a model for prediction of stillbirth after the 28^th^ gestational week in singleton pregnancies of women with overweight or obesity.

**Method:**

This is a register-based cohort study. The first trimester screening database including data from 2006 until 2015 was cross-linked with the Swedish Medical Birth Register and the Swedish Register of Total Population. The final study cohort comprised 145,319 pregnancies, out of which 45,859 pregnancies were complicated by overweight or obesity and without pre-gestational diabetes. There were in total 282 stillbirths. Prediction models for stillbirth in pregnancies with overweight or obesity were constructed based on maternal characteristics, pregnancy complications and biochemical markers. Receiver Operating Characteristic (ROC) and area under curve (AUC) were calculated, based on logistic regression analyses.

**Results:**

The prevalence of stillbirth was 1.6/1000 births and 2.6/1000 births in normal weight and overweight/obese women, respectively. The final predictive model had an AUC of 0.69 (95% CI: 0.64–0.74) with a sensitivity of 28% at a 90% fixed specificity.

**Conclusions:**

It is possible to predict 28% of stillbirths in overweight or obese women, at a false positive rate of 10%. In particular, growth-restricted fetuses are at increased risk of stillbirth.

## Introduction

Stillbirth is most prevalent in low-resource regions of the world, but is also a public health problem in high-resource regions [1, 2] where stillbirth from the 22^nd^ gestational week accounts for the majority of all perinatal deaths [1]. During the past decades the incidence of stillbirth has declined slowly in most high resource countries with an average rate under 5/1000 births and in Sweden 3-4/1000 births [2–4]. However, during the same time period the incidence of neonatal mortality has continuously declined faster and consequently the proportion of perinatal deaths due to stillbirth has increased [4, 5].

Obesity is one of the most frequent modifiable risk factors for stillbirth in high-resource regions [3, 6], with a linearly increased risk of stillbirth with rising maternal body mass index (BMI) [7]. The prevalence of obesity in women in reproductive age is increasing in most countries, including Sweden [4, 8]. In the U.S, more than 30% of women in reproductive age are obese and over 50% are overweight or obese. In most European countries 30–37% of women in reproductive age are overweight or obese [4, 9, 10].

Besides obesity, a number of risk factors for stillbirth have been identified, including for instance previous stillbirth, higher maternal age, smoking, nulliparous women and maternal medical conditions like diabetes, pre-eclampsia, pregnancy induced hypertension, essential hypertension, systemic lupus erythematous (SLE), antiphospholipid syndrome and infections [6, 11–14].

Stillbirth is often accompanied by placental complications and placental dysfunction with fetal growth restriction [1, 3, 6]. Placental dysfunction is reflected in low maternal serum levels of pregnancy associated plasma protein A (PAPP-A) [15, 16]. PAPP-A is a placental enzyme, which releases insulin-like growth factor (IGF) from its´ carrier protein, increasing the proportion of active IGF [17]. IGF and insulin are crucial for fetal growth [17]. It has been proposed that first trimester maternal serum levels of PAPP-A, may predict stillbirth due to impaired placental function [11, 16]. Indeed, low levels of first-trimester PAPP-A in maternal blood, have been found in pregnancies with stillbirth, fetal growth restriction and decreased placental volume [11, 15, 16].

Early identification of pregnancies with an increased risk of stillbirth may open possibilities for monitoring strategies and potential prevention. Given the increasing proportion of pregnancies complicated by obesity and their higher rates of stillbirth, it is of particular interest to develop screening strategies applicable for the obese population. The aim of the present study was to develop a prediction model for stillbirth, possible to use in early pregnancy, in women with overweight or obesity. We hypothesized that a substantial proportion of stillbirths in pregnancies with overweight/obesity could be predicted based on maternal characteristics, maternal first-trimester serum levels of PAPP-A and early fetal growth.

## Material and methods

**Fig 1:**
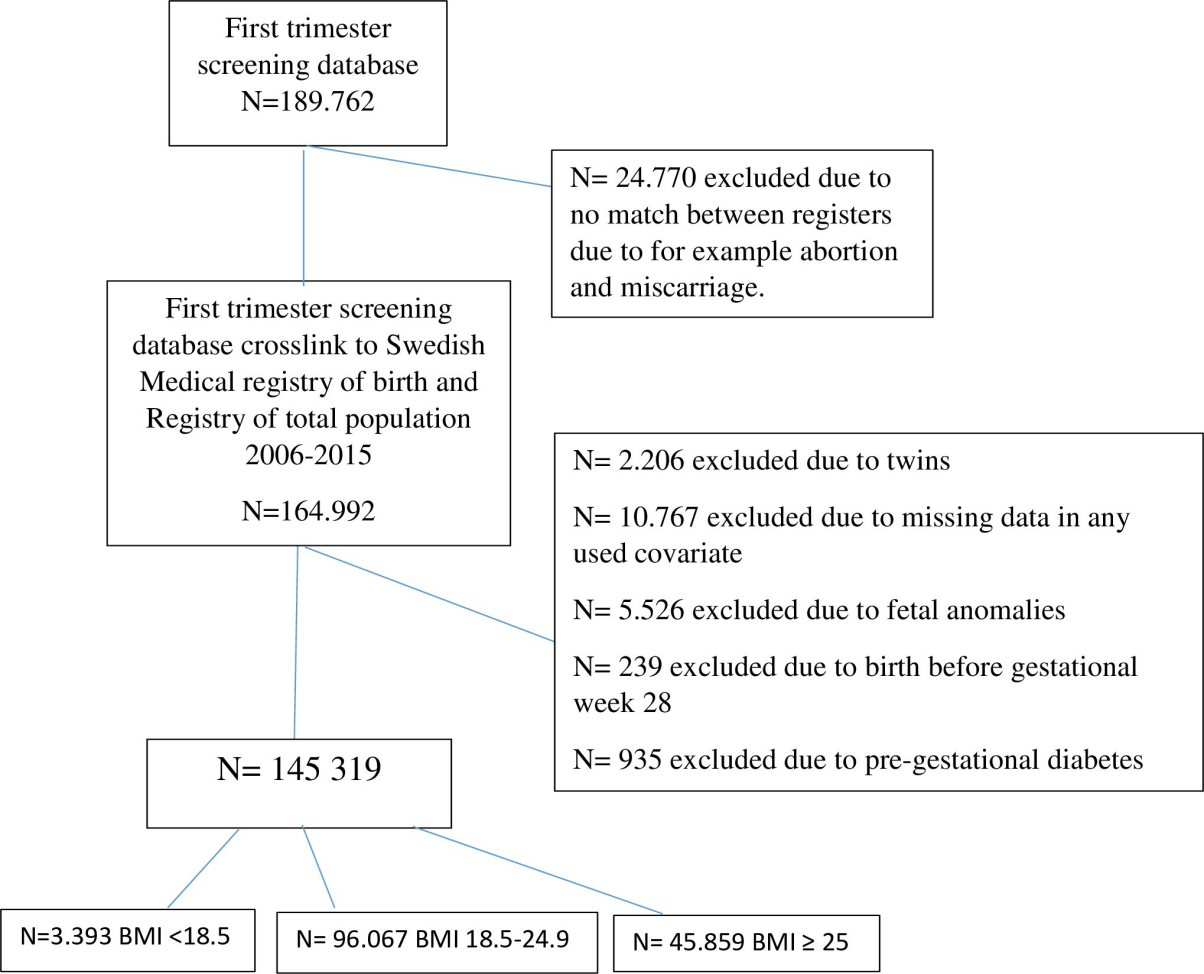
Study cohort flow chart.

This is a registry-based cohort study in Sweden and including all singletons born at gestational week 28 or later, between 2006 and 2015, identified in the first trimester screening database (KUB-database). The first trimester screening database includes data on nuchal translucency scan and levels of PAPP-A in maternal blood. First trimester screening was first implemented in Stockholm, Sweden in 2006. The screening was initially offered to women >35 years of age, or at special request from the woman. However, first trimester screening was gradually offered to all women in Sweden except a few rural counties. The first trimester screening database was cross-linked to the Swedish Medical Birth Register and to the Swedish Register of Total Population. The cross-linking was based on maternal identification number, unique to all citizens in Sweden. The Swedish Medical Birth Register includes information on maternal and infant characteristics as well as maternal and infant diagnoses on 99% of all births in Sweden [18]. Information on the mother’s country of birth, level of education and annual income were obtained from the Swedish Register of Total Population. The three cross-linked registers include information on 164,992 pregnancies. The final study cohort comprised 145,319 pregnancies, after exclusion of pregnancies due to missing data for any of the potential predictors or the outcome (n = 10,767), twins (n = 2,206), fetal anomalies (n = 5,526), delivery before gestational week 28 (n = 239) and due to pre-gestational diabetes (n = 935) (Fig 1). The predictive models were based on all pregnancies to women with BMI ≥ 25 and without pre-gestational diabetes.

### Outcome

Stillbirth from gestational week 28 in singleton pregnancies.

### Predictors

Potential predictors were selected based on the literature or with a plausible biological impact on the risk of stillbirth and included maternal characteristics; BMI, level of PAPP-A, maternal age, smoking status, mother´s region of birth, maternal annual income, educational status, marriage status, maternal drug abuse, parity, previous infant born small for gestational age (SGA), previous pre-eclampsia and previous stillbirth [3, 6]. In addition, pregnancy complications known to be associated with stillbirth were included; diagnosis of antiphospholipid syndrome, diagnosis of SLE, pregnancy induced hypertension, essential hypertension, and gestational diabetes mellitus (GDM) [6, 11], and fetal characteristics; early fetal growth. Small for gestational age (SGA) and pre-eclampsia were not included in the predictive models since these variables are not known in early pregnancy, however included in Table 1.

**Table 1:** Maternal characteristics for women with normal weight compared to women with BMI 25 or above.

	BMI 18.5–24.9 Live born, n = 95,909	BMI 18.5–24.9 Stillborn, n = 158	p-value	BMI >25 Live born, n = 45,735	BMI >25 Stillborn, n = 124	p-value
BMI, median	21.97 (20.7,23.32)	22.1 (20.91,23.34)	0.457	27.78 (26.17,30.49)	28.74 (26.95,32.07)	<0.001
Papp-a, median	1.04 (0.71,1.5)	0.96 (0.63,1.5)	0.113	1 (0.68,1.45)	0.92 (0.66,1.19)	0.005
Age (years)	34 (30,36)	34 (31,37)	0.268	35 (31,37)	36 (33,38)	0.008
Smoking status, yes/no, n (%)	2395 (2.5%)	6 (3.8%)	0.429	2217 (4.85%)	12 (9.68%)	0.022
Born in the nordic countries, n (%)	79125 (82.5%)	134 (84.81%)	0.51	35614 (77.87%)	83 (66.94%)	0.005
Annual income, skr/year, median	243875 (177606,318506)	237834.5 (179927.25,309982.5)	0.403	217789 (155780,278175.5)	211720.5 (125562,269198.5)	0.269
Educational level, median	5 (3,5)	5 (3,5)	0.085	5 (3,5)	3.5 (3,5)	0.087
Mothers living alone, n (%)	1103 (1.17%)	3 (1.92%)	0.615	838 (1.86%)	3 (2.54%)	0.838
Drug abuse, n (%)	346 (0.36%)	0 (0%)	0.927	271 (0.59%)	1 (0.81%)	1
Nulliparous,n (%)	42291 (44.09%)	76 (48.1%)	0.351	16873 (36.89%)	58 (46.77%)	0.029
Previous SGA, n (%)	1193 (1.24%)	2 (1.27%)	1	671 (1.47%)	2 (1.61%)	1
Previous stillbirth, n (%)	150 (0.16%)	1 (0.63%)	0.613	161 (0.35%)	0 (0%)	1
Previous Pre-eclampsia, n (%)	1270 (1.32%)	2 (1.27%)	1	1156 (2.53%)	2 (1.61%)	0.718
Gestational age (weeks)	40 (39,41)	37 (34,40)	<0.001	40 (39,41)	37 (32,40)	<0.001
Antiphosholipid syndrome, n (%)	31 (0.03%)	0 (0%)	1	6 (0.01%)	0 (0%)	1
SLE, n (%)	56 (0.06%)	0 (0%)	1	28 (0.06%)	0 (0%)	1
Pregnancy induced hypertension, n (%)	1235 (1.29%)	2 (1.27%)	1	1297 (2.84%)	2 (1.61%)	0.583
Essential hypertension, n (%)	241 (0.25%)	2 (1.27%)	0.081	456 (1%)	2 (1.61%)	0.813
GDM, n (%)	384 (0.4%)	1 (0.63%)	1	1031 (2.25%)	3 (2.42%)	1
Early fetal growth, days	2 (0,4)	2 (0.5,4)	0.722	2 (0,4)	2 (0,4)	0.222
SGA <10th, n (%)	8488 (8.86%)	56 (36.6%)	<0.001	3180 (6.96%)	50 (41.67%)	<0.001
Pre-eclampsia, n (%)	1963 (2.05%)	4 (2.53%)	0.882	1963 (4.29%)	14 (11.29%)	<0.001

BMI was based on measured weight and self-reported height in the first trimester. PAPP-A level in maternal blood at gestational week 9–13 was entered in the model as multiple of the median (MoM) adjusted for gestational week. Maternal serum-free PAPP-A measurements were performed using immunoassays on the AutoDelfia analyzer in Stockholm (PerkinElmer, Waltham, MA, USA) and Kryptor (Thermo Scientific BRAHMS X, USA) in the other university hospitals. Smoking status was defined as self-reported smoking yes/no at the first antenatal visit. Maternal region of birth was entered in the predictive model, categorized in ten different regions of the world i.e. Sweden, the Nordic countries except Sweden, EU except the Nordic countries, Europe except the Nordic countries and EU, Asia, Africa, South America, North America, Russia and Oceania, however in the tables shown as born in the Nordic countries yes/no (i.e. Sweden, Finland, Norway, Island or Denmark or outside the Nordic countries). Annual income was measured as the woman’s income the year before the infant was born. Maternal educational status was measured as the highest attained level of education the year before the infant was born. Marriage status was self-reported and entered as living together with a partner or living alone. Women with ongoing drug abuse were identified with ICD code O35.5, International Classification of Diseases 10^th^ version. Parity was the number of previous births to each woman. Women with antiphospholipid syndrome or SLE were identified with ICD codes, International Classification of Diseases 10^th^ version: D686 and M32 respectively. Women with a history of pre-eclampsia, a history of pregnancy induced hypertension and women with essential hypertension were identified with ICD codes: O14, O139 and O100 respectively. Women with GDM or a history of GDM were identified with ICD code O244. Assessment of early fetal growth was based on the estimated due date from measures of crown-rump length (CRL) in gestational week 11–14 compared with the estimated due date based on bi-parietal diameter in gestational week 18–20, giving a number of days where negative values indicate a decelerated early fetal growth. SGA was defined as birthweight < the 10^th^ percentile for gestational age according to the general Swedish fetal population [19].

### Statistical analysis

Frequencies of all potential predictors were compared between women with a stillborn fetus and women with a live born fetus and stratified by maternal BMI (18.5–24.9 or BMI ≥ 25). Predictors measured on a continuous scale were presented as medians with interquartile range (IQR) and categorical predictors as numbers and proportions. Comparisons between the continuous predictors were done with Wilcoxon ranksum test and with chi-square test for the categorical predictors. The following predictors, identified in the univariable analyses included; BMI, PAPP-A, maternal age, smoking status, the mother’s region of birth and parity. The prediction model was based on risk estimates from the logistic regression analysis. The final model for prediction of stillbirth in early pregnancy included predictors significantly associated with stillbirth in the multivarible regression model, (e.g. BMI, PAPP-A, maternal age, smoking status, the mother’s region of birth and parity).

The confidence intervals and p-values were corrected for multiple births by the same woman by using a sandwich estimate of variance [20]. Receiver Operating Characteristic (ROC) curves and area under curve (AUC) were calculated.

Strong interactions between the predictors may impact the performance of the main models. Accordingly, the analyses above were re-run including all possible two-way interactions between the predictors.

The predictive capacity of the model was investigated with a cross-validation procedure. Specifically, the dataset was randomly split in two parts. The first part was used to fit the model and the second part was used to estimate the AUC. This procedure was repeated 1000 times, and the mean (over these 1000 repetitions) AUC were compared with the AUC obtained without cross-validation.

The median BMI and prevalence of stillbirth were calculated for pregnancies with missing data on any of the predictive variables.

Statistical differences were considered significant given a p-value <0.05.

All statistics were performed using R cran, package pROC [21] and drgee [22].

Ethics approval for this study was obtained from the Regional Research Ethics Committee at Karolinska institute in Stockholm, Sweden (2011/1006-31/4 with amendment 2016/952-32 and 2018/1066-32). Data were fully anonymized before access. The Ethics Committee did not require written informed consent. The Ethics Committee prohibit data to be publicly available. However, data will be shared after an approval from the Regional Research Ethics Committee.

## Result

The study cohort included 145,319 pregnancies. There were 45,859 pregnancies from 41,010 unique women with overweight or obesity and without pre-gestational diabetes. The prevalence of stillbirth was 2.6/1000 births in pregnancies complicated by overweight/obesity and 1.6/1000 births in women with BMI within the normal range (i.e. BMI 18.5–24.9 kg/m^2^). The median gestational age at stillbirth was 37 weeks both in pregnancies complicated by overweight or obesity and in normal weight pregnancies. Maternal characteristics are described in Table 1, comparing women with BMI 18.5–24.9 and women with BMI ≥ 25. For the whole group of pregnancies with stillborn fetuses, the median PAPP-A levels were lower and the proportion of SGA markedly increased compared to pregnancies with live born infants. The potential predictors BMI, smoking status, maternal age, parity, maternal region of birth and pre-eclampsia were all significantly associated with stillbirth among women with overweight or obesity, however they were not associated with stillbirth in pregnancies of women with normal weight.

**Fig 2:**
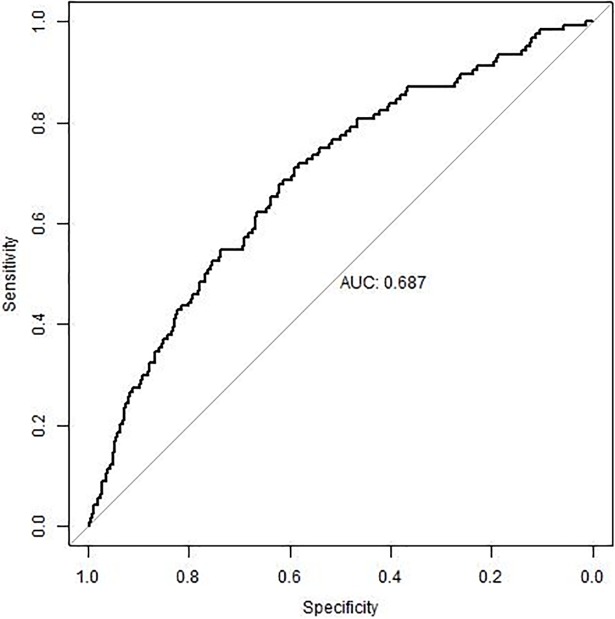
Prediction of stillbirth. The final predictive model of stillbirth for overweight or obese women in early pregnancy had a ROC and AUC of 0.69 (95% CI 0.64–0.74) with 28% sensitivity at 90% fixed specificity and 44% sensitivity at 80% fixed specificity.

The estimated odds-ratios from the logistic regression are presented in Table 2. The area under the curve (AUC) summarizes the test performance in terms of sensitivity-specificity trade-off; the higher sensitivity, at each fixed specificity, the higher AUC. The AUC for the ROC curve of the final predictive model was 0.69 (95% CI 0.64–0.74) (Fig 2). The sensitivity was 28% at a fixed 90% specificity. The AUC after the cross-validation procedure for the final predictive model was 0.65. In addition, the model was re-run with BMI as a categorical predictor see, S1 Fig and S1 Table.

**Table 2:** Estimated odds-ratios.

Logistic regression, Stillborn infants, women with BMI 25 or above			
	Live-born	Stillborn OR (95% CI)	p-value
BMI, (per unit)	Ref	1.06 (1.03, 1.10)	< 0.001
PAPP-A, (IU/L)	Ref	0.60 (0.44, 0.83)	0.002
Age, (years)	Ref	1.05 (1.01, 1.09)	0.008
Smoking status, (yes/no)	Ref	2.25 (1.24, 4.08)	0.007
Country of birth, (Nordic country yes/no)	Ref	0.58 (0.40, 0.84)	0.004
Parity, (per child)	Ref	0.79 (0.64, 0.97)	0.02

Estimated odds-ratios and p-values from the logistic regression analyses for all predictors included in the final predictive model.

The levels of PAPP-A were lower among the overweight and obese women compared to the normal weight women (p-value < 0.001).

The number of missing data was low, under 0.5% for each of the predictors. The median BMI for pregnancies with missing data on any predictor was 28.0 kg/m^2^ compared with 27.8 kg/m^2^ in pregnancies with data on all covariates (p-value 0.16). The incidence of stillbirth among pregnancies with missing data on any predictor was 3/1000 compared with 2/1000 in pregnancies with data on all covariates (p-value 0.002).

The AUC slightly increased when models were re-run taking all possible two-way interactions into account. However, the AUC decreased after cross-validation with all possible two-way interactions taken into account, S2 Fig.

## Discussion

This prospective cohort study presents a clinical model for early prediction of stillbirth in women with overweight or obesity. The predictive capacity of the final prediction model was reasonable with an AUC of 0.69 (95% CI: 0.64–0.74) and a sensitivity of 28% at 90% fixed specificity, see Fig 2. A predictive model with an AUC 0.70–0.80 is commonly interpreted as fairly good, AUC 0.8–0.9 as good and AUC 0.9–1 as excellent [23]. However, when interpreting the strength of a prediction it is also important to be aware of the prevalence or incidence of the outcome and the consequences of false negative or false positive results. Stillbirth is a rare outcome, however a severe complication. A higher proportion of cases would be possible to identify with a decreased specificity, hence an increased sensitivity. A decreased specificity will increase the number of false positive results; however, this might be acceptable since stillbirth is such a severe outcome. Our final predictive model has for example 44% sensitivity at 80% fixed specificity; see Fig 2.

Strengths of our study include a large sample size with data on 45,859 pregnancies complicated by overweight or obesity, without pre-gestational diabetes. Information on maternal characteristics, diagnoses during pregnancy and delivery and levels of early biochemical markers in maternal blood were prospectively collected. It has been shown that prospectively collected self-reported information on smoking status is valid in Sweden [24].

Limitations of our study include the possibility of selection bias since the first trimester screening was initially only offered to women aged 35 years or older or at special request. Furthermore, the first trimester database covers large parts of Sweden but not the whole country, and with focus on areas around the larger cities. The prevalence of female obesity is lower in our study population than in the general Swedish population as well as the incidence of stillbirth [4]. The characteristics of the study population may have affected the results in at least two ways. First, since predictions generally become better when the predictors have high variability (e.g. include both low and high values), the relatively low variability of predictors in our study population are likely to have biased the AUCs downwards (towards 0.5). Second, if the associations between the predictors and the outcome (stillbirth) differ between our study population and the general obstetric population, then our estimated AUCs may be biased as well; a weaker association in our study population would have biased the AUCs downwards and a stronger association would have biased the AUCs upwards (towards 1). However, it is not obvious whether the predictor-outcome associations are different in our study population compared with the general obstetric population.

Although stillbirth is defined as infants born dead after the 22^nd^ gestational week in Sweden, we chose stillbirth after the 28^th^ gestational week as outcome since the value in a predictive model is to be able to take preventive action. It is harder to find potential preventive strategies and reasonable acting for the very earliest stillbirths.

To our knowledge this is the first attempt to make a model for prediction of stillbirth in pregnancies complicated by overweight or obesity. However, a few previous studies report models for early prediction of stillbirth in populations with mixed BMI [11, 16]. As the etiology of stillbirth is complex the performance of the predictors most likely vary between different high-risk groups [16]. For instance, PAPP-A and uterine artery pulsatility index have been proposed as good predictors of stillbirth in pregnancies complicated by impaired placental function [11, 16]. With pulsatility index in both ductus venosus and the uterine artery the predictive capacity is likely increased [11]. SGA is strongly associated with stillbirth. In the current study, we only had information on estimated fetal growth between the 11^th^ and 20^th^ week. The estimate of early fetal growth was not significantly associated with stillbirth in the multivariate analysis, however inclusion of data on early fetal growth increased the AUC of the final predictive model. Accordingly, inclusion of SGA in the predictive model improved its performance (AUC = 0.79 (95% CI: 0.75–0.83). Thus, it is likely that the predictive capacity of our model would be improved if estimated fetal weight in gestational week 28–32 was included in the model.

We found significantly lower levels of PAPP-A in obese women compared with normal weight women. Maternal diabetes has also been associated with decreased levels of maternal PAPP-A [25]. Abnormal glucose metabolism may negatively impact placentation by influencing trophoblast invasion [26] and it has been suggested that levels of PAPP-A may reflect the degree of maternal glucose intolerance [25]. Thus, it is possible that the lower levels of PAPP-A observed in women with diabetes or obesity reflects a suboptimal placentation [25]. Signs of placental immaturity and placental changes implicating an aging placenta are more often seen in pregnancies with obesity [27], and may also contribute to the increased risk of fetal demise.

The mechanisms behind the increased risk of stillbirth in pregnancies of overweight or obese women is unclear [28]. However, placental inflammation and dysfunction as well as metabolic and hormonal changes associated with obesity have been suggested to increase the risk of stillbirth [28]. Maternal hyperglycemia in pregnancies complicated by pre-gestational or severe gestational diabetes increases risk of stillbirth [29]. Maternal hyperglycemia may lead to fetal hyperinsulinemia and increased demand for oxygen [30]. In case of impaired placental function, this mechanism might contribute to increased risk of fetal hypoxia and death. One might speculate that elevated maternal glucose levels also within the normal range (i.e. below the threshold for diabetes) may have a negative impact on fetal metabolism and oxygen supply. In the current study, GDM was not identified as an independent risk factor for stillbirth. However, earlier studies have shown an association between GDM and increased risk of stillbirth after the 28^th^ gestational week, in a population of women with different BMI [31].

It is interesting to speculate on possible preventive strategies to reduce the risk of fetal loss in obese pregnancies. Surveillance of fetuses in pregnancies complicated by overweight or obesity differs throughout Sweden and between countries. However, we are not aware of guidelines recommending extended fetal surveillance during pregnancy in obese women [32, 33] In Sweden, there are no routine ultrasound after the organ screening ultrasound in gestational week 18–20. Given the increased risk of stillbirth in obese pregnancies and in particular in SGA infants [28, 32, 34, 35], ultrasound assessing fetal growth at 28–32 weeks of pregnancy could be of value.

## Conclusion

An early prediction of stillbirth in pregnancies of overweight or obese women, based on predictors accessible for the clinician, can possibly detect 28% of cases at 90% fixed specificity. Results from the predictive model may be helpful in discussing risks and pregnancy surveillance with the pregnant woman and her partner. Further development of the predictive model by including data on estimated fetal weight and other potential predictors as well as analysis of the cost-benefit ratio of a third trimester ultrasound are important areas for future studies.

## Supporting information

S1 Fig If BMI is included as a categorical predictor instead of a continuous predictor the AUC was 0.68 (95% CI: 0.63–0.73), S1 Fig. When the model was re-run with cross-validation the AUC was 0.63. When BMI is included in the model as a categorical predictor the AUC decreases slightly both before cross-validation and after cross-validation.
(DOCX)

S2 Fig The AUC of the final predictive model taking all possible two-way interactions into account was 0.72 (95% CI: 0.68–0.76), a slight increase. However, the AUC after cross-validation for the final model with all possible two-way interactions taken into account decreased to 0.61. The decreased AUC after cross-validation indicate that the slightly increased AUC with two-way interactions was due to an over-fit to the data.
(DOCX)

S1 Table The estimated odds were slightly changed when BMI was included as a categorical predictor instead of a continuous. Estimated odds-ratios and p-values from the logistic regression for all predictors in the final predictive model, with BMI as a categorical predictor.
(DOCX)
