# Supplementary material for: Prediction of stillbirth in women with overweight or obesity—A register-based cohort study
Source: PLoS One. 2018 Nov 19;13(11):e0206940. doi: 10.1371/journal.pone.0206940 (PMC6242307; doi:10.1371/journal.pone.0206940)
Supplement: S1 Fig — When the model was re-run with cross-validation the AUC was 0.63. When BMI is included in the model as a categorical predictor the AUC decreases slightly both before cross-validation and after cross-validation. (DOCX) [file pone.0206940.s001.docx]

Supporting information

**S1 Fig**


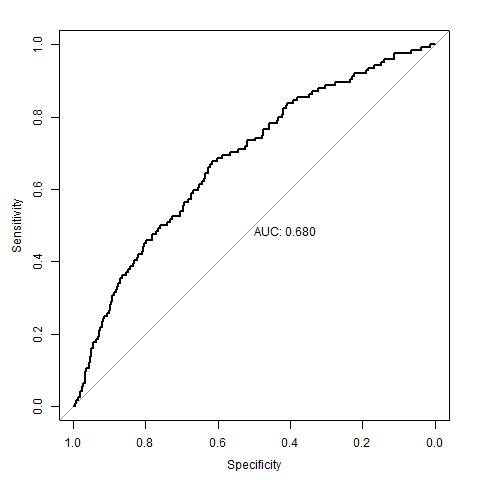


**S1 Fig**

If BMI is included as a categorical predictor instead of a continuous predictor the AUC was 0.68 (95% CI: 0.63-0.73), S1 Fig. When the model was re-run with cross-validation the AUC was 0.63. When BMI is included in the model as a categorical predictor the AUC decreases slightly both before cross-validation and after cross-validation.
