# Supplementary material for: Prediction of stillbirth in women with overweight or obesity—A register-based cohort study
Source: PLoS One. 2018 Nov 19;13(11):e0206940. doi: 10.1371/journal.pone.0206940 (PMC6242307; doi:10.1371/journal.pone.0206940)
Supplement: S2 Fig — However, the AUC after cross-validation for the final model with all possible two-way interactions taken into account decreased to 0.61. The decreased AUC after cross-validation indicate that the slightly increased AUC with two-way interactions was due to an over-fit to the data. (DOCX) [file pone.0206940.s002.docx]

**S2 Fig**


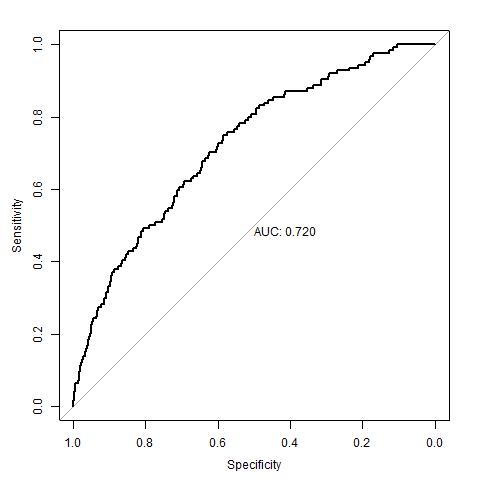


**S2 Fig**

The AUC of the final predictive model taking all possible two-way interactions into account was 0.72 (95% CI: 0.68-0.76), a slight increase. However, the AUC after cross-validation for the final model with all possible two-way interactions taken into account decreased to 0.61. The decreased AUC after cross-validation indicate that the slightly increased AUC with two-way interactions was due to an over-fit to the data.
