# Supplementary material for: Prediction of stillbirth in women with overweight or obesity—A register-based cohort study
Source: PLoS One. 2018 Nov 19;13(11):e0206940. doi: 10.1371/journal.pone.0206940 (PMC6242307; doi:10.1371/journal.pone.0206940)
Supplement: S1 Table — Estimated odds-ratios and p-values from the logistic regression for all predictors in the final predictive model, with BMI as a categorical predictor. (DOCX) [file pone.0206940.s003.docx]

**S1 Table**

**S1 Table:** Estimated odds-ratios

| Logistic regression, Stillborn infants, women with BMI 25 or above | | | |
| --- | --- | --- | --- |
|  | Live-born | Stillborn OR (95% CI) | p-value |
| BMI 25-29.9 | Ref | Ref |  |
| BMI ≥ 30 | Ref | 1.48 (1.02, 2.14) | 0.04 |
| PAPP-A, (IU/L) | Ref | 0.60 (0.44, 0.82) | 0.002 |
| Age, (years) | Ref | 1.05 (1.01, 1.09) | 0.008 |
| Smoking status, (yes/no) | Ref | 2.29 (1.26, 4.17) | 0.007 |
| Country of birth, Nordic country (yes/no) | Ref | 0.58 (0.40, 0.85) | 0.005 |
| Parity, (per child) | Ref | 0.79 (0.64, 0.97) | 0.03 |

The estimated odds were slightly changed when BMI was included as a categorical predictor instead of a continuous. Estimated odds-ratios and p-values from the logistic regression for all predictors in the final predictive model, with BMI as a categorical predictor.
